# Systemic contact dermatitis to diclofenac associated with contact sensitivity to piroxicam

**DOI:** 10.1186/2045-7022-4-S3-P84

**Published:** 2014-07-18

**Authors:** Nicola Wagner, Maurizio Podda

**Affiliations:** 1 Department of Dermatology, Clinical Centre Darmstadt, Germany; 2 Clinical Centre Darmstadt, Department of Dermatology, Germany

## 

A 15-year-old boy developed contact dermatitis after a foot injury treated with diclofenac. Two years later he ingested diclofenac p.o. for pain relief after exercise. The following day he showed a generalized eczema, fading away during the next two weeks. Subsequently paracetamol was well tolerated but had no sufficient analgetic effect. Patch testing was performed with different non-steroidal drugs (ibuprofen, acetylic acid, indometacin, celecoxib, and piroxicam) in concentrations of 1% , 5 % and 10 % diluted in petrolatum, confirming contact sensitivity to diclofenac. Piroxicam showed a positive patch test reaction, although the patient denied any former contact to the substance. Cross-reactivity can be excluded as both analgetics belong to chemically different groups of NSAID. Ibuprofen was orally exposed and well-tolerated. Recent data have demonstrated that delayed hypersensitivity reactions due to drug-specific T-cells may be subclassified by their cytokine and chemokine releasing profile evolving into different clinical manifestations. Activation of monocytes (type IVa), eosinophils (type IVb) and neutrophils (type IVd) may dominate the reaction, cyctotoxic CD4(+) or CD8(+) T-cells seem to play a part in all delayed-type reactions. As contact sensitivity to piroxicam is rarely reported, possible mechanisms are discussed.

